# Reduction of Microglial Reactivity by Cannabidiol: Preliminary Data Obtained in an Astrocyte–Microglia Co-Culture Model of Inflammation

**DOI:** 10.3390/ph19091342

**Published:** 2026-08-24

**Authors:** Laura Schönfelder, Shaoning An, Peter Reusch, Pedro M. Faustmann, Timo Jendrik Faustmann, Fatme S. Ismail

**Affiliations:** 1 Department of Neuroanatomy and Molecular Brain Research, Medical Faculty, Ruhr University Bochum, 44801 Bochum, Germany; laura.schoenfelder@ruhr-uni-bochum.de (L.S.); shaoning.an@ruhr-uni-bochum.de (S.A.); pedro.faustmann@ruhr-uni-bochum.de (P.M.F.); 2 Department of Clinical Pharmacology, Medical Faculty, Ruhr University Bochum, 44801 Bochum, Germany; peter.reusch@ruhr-uni-bochum.de; 3 Department of Psychiatry and Psychotherapy, Medical Faculty, Heinrich Heine University, 40629 Düsseldorf, Germany; timo.faustmann@uni-duesseldorf.de

**Keywords:** cannabidiol, pregabalin, microglia, inflammation, astrocytes

## Abstract

**Background/Objectives**: Glia-mediated inflammation contributes to a wide range of central nervous system (CNS) disorders, including epilepsy. Pregabalin (PGB) and cannabidiol (CBD) are CNS-acting drugs prescribed for various neuropsychiatric conditions, especially seizures. This study investigated the effects of PGB and CBD on glial properties in an astrocyte–microglia co-culture model of inflammation. **Methods:** Physiological (M5, containing 5–10% microglia) and pathological inflammatory astrocyte–microglia co-cultures (M30, containing 30–40% microglia) were collected from the postnatal brain hemispheres of Wistar rats (P0-P2) according to an established protocol and treated with different concentrations of PGB (3, 10, 30 and 60 µg/mL) for 24 h or CBD (50, 500 and 1000 ng/mL) for 1 h or 24 h. Metabolic activity was assessed by the MTT assay. Microglial phenotypes and astroglial connexin (Cx)43 expression were detected by immunocytochemistry. **Results:** In M5 co-cultures, short-term incubation (1 h) with high concentrations (1000 ng/mL) of CBD significantly reduced glial viability (*p* < 0.05), while no significant changes were observed in M30 co-cultures. After 24 h of incubation, M5 cultures exhibited a significant increase in metabolic activity at 50 ng/mL (*p* < 0.0001) and 500 ng/mL (*p* < 0.05), and a reduction at 1000 ng/mL (*p* < 0.05), suggesting impaired viability at high concentrations under physiological conditions. The distribution of microglial phenotypes in physiological M5 co-cultures incubated with CBD remained unchanged. In M30 co-cultures, CBD incubation for 1 and 24 h significantly reduced microglial activation and promoted a shift from reactive, phagocytic to homeostatic, ramified microglial phenotype (*p* < 0.05, *p* < 0.01, *p* < 0.0001). In contrast, PGB did not affect glial cell viability, microglial phenotypes or Cx43 expression in physiological and inflammatory co-cultures, indicating that the mechanisms of action of PGB probably do not include modulation of glial cells in vitro. **Conclusions:** The inhibition of microglial reactivity by CBD suggests potential positive effects on the neuroinflammatory component involved in the pathogenesis of CNS disorders such as epilepsy.

## 1. Introduction

The treatment of neuropsychiatric disorders has become increasingly challenging in recent years because pharmacological therapeutic options are limited [1]. Pregabalin (PGB) and cannabidiol (CBD) are both substances used in the treatment of epilepsy but refer to different pathways of action. Since both are also widely used in the treatment of other neuropsychiatric disorders, a comparison of their effects on glia-mediated inflammatory mechanisms is of interest. PGB is approved by the Food and Drug Administration (FDA) for the treatment of neuropathic pain (diabetic peripheral neuropathy, postherpetic neuralgia, spinal cord injury) and fibromyalgia and as adjunctive therapy for patients with epilepsy (partial-onset seizures), and by the European Medicines Agency (EMA) for neuropathic pain, epilepsy and generalized anxiety disorder. Further, possible off-label uses include bipolar disorder, insomnia and social anxiety [2,3,4]. PGB is structurally similar to the inhibitory neurotransmitter gamma-aminobutyric acid (GABA), but does not bind to GABA receptors. In the central nervous system (CNS), it has a binding affinity for the presynaptic voltage-gated calcium channels (alpha-2-delta subunit) and reduces the release of excitatory neurotransmitters that may be responsible for the anticonvulsant and analgesic effects of PGB [4]. So far, the multifaceted mechanisms of therapeutic action of PGB offer new approaches to understanding pathological mechanisms in neuropsychiatric disorders.

CBD is a cannabinoid derived from the *Cannabis sativa L.* plant [5,6]. CBD represents the active ingredient in drugs (Epidiolex^®^/Epidyolex^®^) that are approved by the FDA for the treatment of seizures associated with Lennox–Gastaut syndrome (LGS), Dravet syndrome (DS) or tuberous sclerosis complex (TSC) in patients ≥1 year of age, and by the EMA for adjunctive use (in combination with clobazam) to treat seizures associated with LGS and DS, as well as for adjunctive use to treat seizures associated with TSC in patients ≥2 years of age [7,8,9,10,11]. Additionally, CBD has received approval for the relief of moderate to severe spasticity in multiple sclerosis (MS), the most common chronic demyelinating autoimmune CNS disease. In the form of the drug nabiximols (Sativex^®^), which contains tetrahydrocannabinol (THC) in addition to CBD, it is used to treat this condition. However, side effects such as dizziness, fatigue, or even depression may occur [12]. Beyond its symptomatic use in MS-associated spasticity, preclinical studies suggest that CBD may exert immunomodulatory and neuroprotective effects in experimental models of demyelinating disease. In experimental autoimmune encephalomyelitis (EAE), a commonly used model of MS, CBD treatment has been associated with reduced clinical disease severity, inflammatory-cell infiltration, demyelination and axonal damage. In vitro, incubation of encephalitogenic cells with CBD reduced their viability, which was associated with increased early apoptosis and reactive oxygen species (ROS) generation, as well as decreased production of interleukin-6 (IL-6). These findings suggest that the direct effects of CBD on pathogenic immune cells may contribute to its protective effects in EAE [13].

CBD is also being studied for other indications such as treatment of focal-onset seizures—e.g., in the EpiFOS exploratory study (NCT07233239). However, the precise mechanisms by which CBD exerts its anticonvulsant effect in humans are yet unknown. Experimental studies have shown anticonvulsant and neuroprotective effects of CBD in several seizure and epilepsy models, such as pentylenetetrazole (PTZ)-induced seizures, pilocarpine-induced status epilepticus and corneal or limbic kindling paradigms, with reductions in seizure development and severity, and attenuation of neuroinflammation and neuronal loss. More specifically, in PTZ-induced seizure models, CBD increased seizure latency, reduced seizure severity and duration, and delayed the progression of PTZ kindling. These effects were accompanied by prevention of seizure-associated increases in prefrontal cortical IL-6 and modulation of mechanisms involving endocannabinoid and TRPV1 signaling [14]. In pilocarpine-induced seizure models, CBD reduced the occurrence of severe seizures and seizure-related mortality, supporting an anticonvulsant and potentially neuroprotective effect in this paradigm [15]. In corneal and limbic kindling models, CBD reduced seizure expression and severity; in the limbic kindling model, it also reduced seizure duration and amplitude and increased the seizure threshold, although the magnitude of these effects was dependent on the model and dose [16,17]. The mechanisms underlying these effects are likely to be multimodal. It has been suggested that the reported inverse agonist activity of CBD at the cannabinoid receptor 2 (CB2 receptor) may contribute to its anti-inflammatory properties [18]. However, these effects may also involve other endocannabinoid-related and non-endocannabinoid signaling pathways. The exact molecular and cellular targets mediating the antiseizure effects of CBD in humans have yet to be fully elucidated [14,16,19,20,21].

Interestingly, effects on the CB1 and CB2 receptor, but additionally an involvement of the 5HT(1A) receptor, were discussed as modulating inflammation in a hypoxic–ischemic model [22,23]. Although so far not approved, CBD represents an interesting approach for the treatment of mood disorders and their associated inflammatory mechanisms.

Furthermore, both PGB and CBD are discussed in the field of substance use disorders (SUD)—CBD for the treatment of SUD and PGB more related to consumption together with other substances [24,25,26].

In recent years, in addition to neurons, the involvement of non-neuronal glial cells (such as microglia and astrocytes) in epilepsy, bipolar disorder, anxiety disorders and neuropathic pain has been recognized and offers new diagnostic and therapeutic perspectives [27,28,29,30,31,32]. Microglia are resident immune effector cells of the CNS, which are morphologically, immunophenotypically and functionally related to cells of the monocyte/macrophage lineage, and respond to inflammatory conditions with proliferation, cytokine release and morphological changes from a homeostatic ramified type over an intermediate type to a rounded phagocytic type [33,34]. Astrocytes are the main type of non-neuronal cells in the CNS, supporting brain homeostasis, forming part of the blood–brain barrier and stabilizing neuronal networks [35,36]. Connexin 43 (Cx43) is the main gap-junctional (GJ) protein in astrocytes, contributing to direct cell-to-cell communication and intercellular exchange [37,38,39]. Enhanced gap junctional communication (GJC) not only between neurons but also between astrocytes has been associated with seizure activity, confirming the involvement of glial cells in seizure generation [39,40,41]. Moreover, increased GJC and upregulation of Cx43 have been linked to the development of neuropathic pain in neuropathic pain models [42,43,44]. Further studies regarding epilepsy showed that inflammation can act as both a cause and a consequence of epileptic seizures, and inflammation is involved in the pathological mechanisms of epilepsy [45,46,47]. Additionally, glial cells and inflammatory conditions were found to be involved in neuropathic pain and psychiatric disorders [48,49,50,51,52,53].

An astrocyte–microglia co-culture model developed by Faustmann et al. (2003) makes it possible to study pharmacological effects under physiological M5 (containing 5–10% microglia) and pathological M30 conditions (containing 30–40% microglia) [34]. Antiseizure medications (ASMs) such as lacosamide (LCM), lamotrigine (LTG), topiramate (TPM), levetiracetam (LEV), brivaracetam (BRV), valproic acid (VPA), gabapentin (GBT), phenytoin (PHE), carbamazepine (CBZ), zonisamide (ZNS), and tiagabine (TGB) have been used to investigate effects on glial cells in terms of inflammation, simulating severe diseases of the CNS such as epilepsy [54,55,56,57,58,59,60].

Taking together, PGB and CBD are interesting substances in the field of neuropsychiatry, both for the approved field of epileptic seizures and for psychiatric diseases. A possible overlapping mechanism of action could be voltage-gated calcium channels [61,62].

The increasing evidence of the involvement of astrocytes and microglia in epilepsy and other neuropsychiatric conditions highlights CBD and PGB as promising CNS drugs to study in our co-culture model. Here, we investigated the effects of CBD and PGB on glial cell viability, microglial phenotypes and expression of the GJ protein Cx43 as well as functional GJ coupling in an astrocyte–microglia co-culture model of inflammation.

## 2. Results

### 2.1. Effects of PGB and CBD on Glial Cell Viability

**Figure 1:**
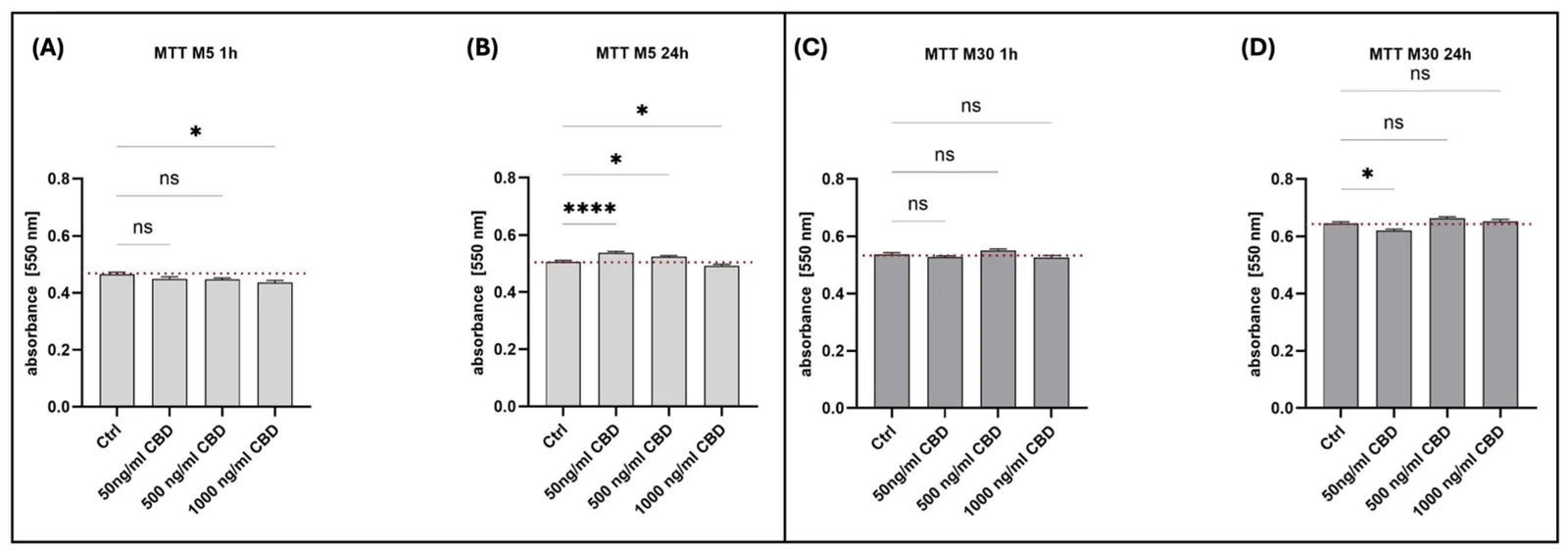
MTT assay results of M5 and M30 co-cultures after incubation with CBD (50, 500, 1000 ng/mL) for either 1 h or 24 h. (**A**) One hour of CBD incubation showed a significant reduction in absorbance for M5 at 1000 ng/mL (*p* < 0.05), while no significant changes (**C**) were observed for M30. (**B**) After 24 h, M5 cultures exhibited a significant increase in absorbance at 50 ng/mL (*p* < 0.0001) and 500 ng/mL (*p* < 0.05), with a reduction at 1000 ng/mL (*p* < 0.05). (**D**) In M30, a significant reduction in absorbance was observed only at 50 ng/mL (*p* < 0.05), with no significant changes at higher concentrations. Data represent means ± SEM. Normality was assessed using the D’Agostino-Pearson test; depending on data distribution, one-way ANOVA or the Kruskal–Wallis/Dunn post hoc test was applied. Ctrl: control; CBD: cannabidiol; ns: not significant; * *p* < 0.05, **** *p* < 0.0001; *n* = 24.

To investigate the impact of CBD on glial cell viability and metabolism, MTT assays were performed on both M5 and M30 astrocyte–microglia co-cultures, measuring absorbance at 550 nm after incubation with various concentrations of CBD for 1 or 24 h. Results for both co-cultures are summarized in Figure 1.

Across both cell models, short-term (1 h) CBD incubation had minimal impact on absorbance, except for a significant reduction seen in M5 cultures exposed to 1000 ng/mL. After 24 h, M5 cultures showed increased absorbance at lower CBD concentrations (50 and 500 ng/mL) and decreased absorbance at 1000 ng/mL, indicating dose- and time-dependent effects. In contrast, M30 co-cultures were largely unaffected, except for a significant decrease in absorbance at 50 ng/mL after 24 h. No other differences reached statistical significance. These findings suggest that M5 and M30 co-cultures differ in their metabolic response to CBD, particularly after prolonged exposure.

Physiological M5 and pathological M30 astrocyte–microglia co-cultures were incubated with 3, 10, 30 and 60 µg/mL PGB for 24 h. Incubation with PGB at various concentrations did not lead to significant changes in measured absorbance regarding glial cell viability under both physiological and pathological conditions (Figure S1).

### 2.2. Glial Cell Numbers and Microglial Phenotypes After Incubation with CBD or PGB

#### 2.2.1. CBD Significantly Reduced Microglial Reactivity Under Pathological, Inflammatory Conditions

Quantification of cell nuclei using DAPI staining revealed model- and dose-dependent effects of CBD. In the M5 (physiological) co-cultures, a 1 h incubation with 1000 ng/mL CBD led to a significant increase in cell number (mean: 90 nuclei, control: 76; *p* < 0.001). After 24 h, 500 ng/mL CBD increased the mean cell number to 88 compared to 82 in controls (*p* < 0.05). In contrast, neither 1 h nor 24 h CBD incubation altered the total cell number in the pathological M30 co-cultures at any concentration, with cell numbers remaining stable across all groups. These results indicate that CBD may modestly elevate cell numbers in M5 co-cultures at specific concentrations and time points but does not affect cell proliferation or survival in M30 co-cultures under these experimental conditions (Figure S2).

Microglia were quantified using ED-1 staining. In M5 co-cultures, the average number of microglial cells at 1 h ranged from 5 to 6 cells per field, with no significant change across CBD concentrations. After 24 h, the highest CBD concentration (1000 ng/mL) yielded a modest but significant increase in microglial cell numbers (*p* < 0.05). In contrast, M30 co-cultures exhibited consistent microglial cell numbers regardless of CBD concentration or incubation time, indicating no acute or delayed effects of CBD on microglial cell numbers in this model (Figure S3). Additionally, the immunocytochemical expression of CNR/CB2 (cannabinoid receptor 2) was detected in all microglial phenotypes (Figure S4).

**Figure 2:**
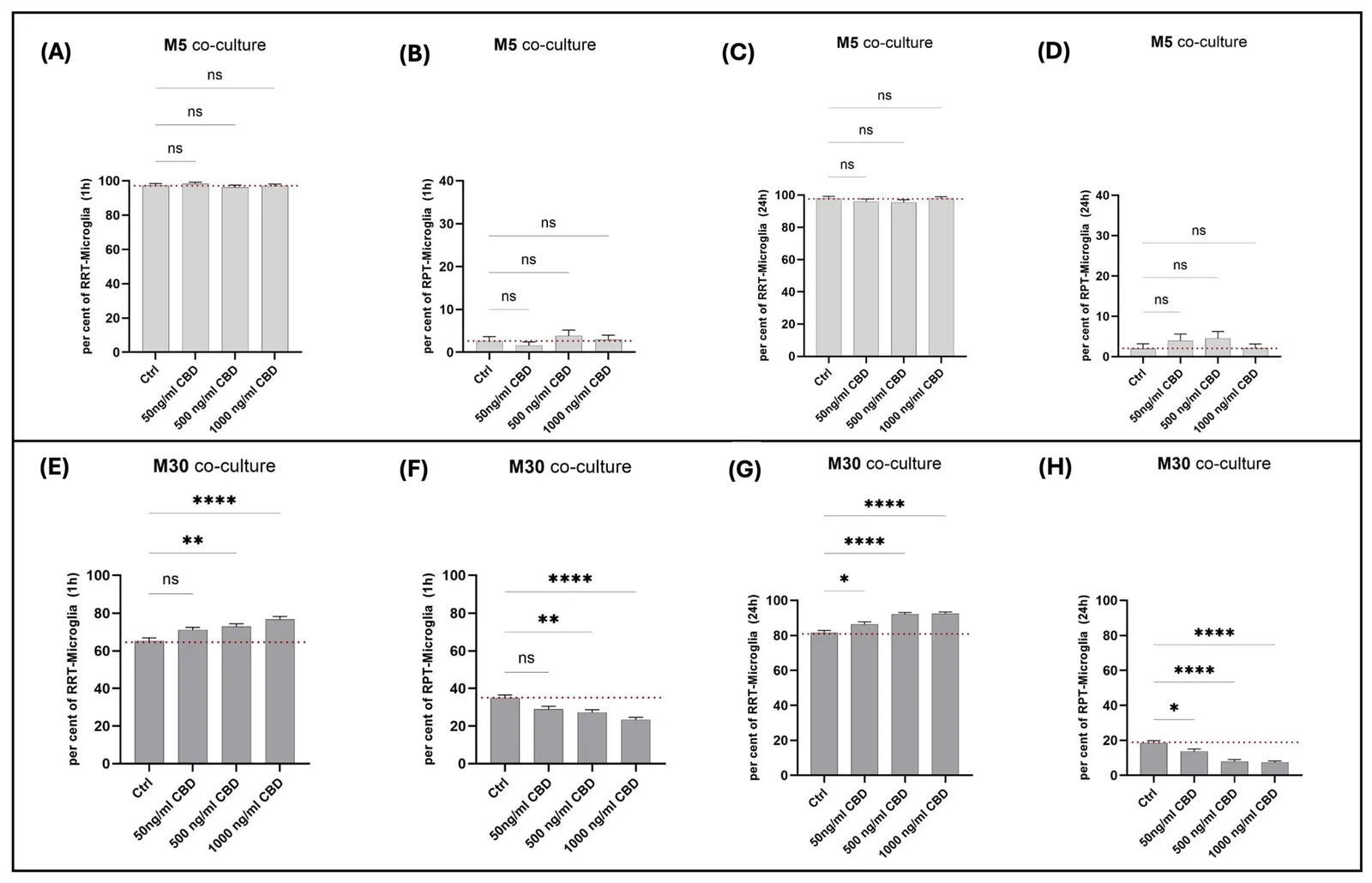
Phenotype distribution of microglia in M5 and M30 co-cultures after one or 24 h of CBD incubation. No significant changes in the proportion of homeostatic ramified (RRT) (**A**,**C**) or activated (RPT) (**B**,**D**) microglia were found in the M5 group at any timepoint or concentration. In the M30 group, one-hour and 24 h incubations with increasing concentrations of CBD resulted in a dose-dependent increase in the percentage of homeostatic ramified RRT microglia (**E**,**G**) and a corresponding decrease in activated RPT microglia (**F**,**H**), with most changes significant at ≥50 ng/mL CBD. Data: mean ± SEM. Normality was assessed using the D’Agostino–Pearson test; normally distributed datasets were analyzed by one-way ANOVA, and non-normally distributed datasets were analyzed by Kruskal–Wallis with Dunn’s post hoc test. Ctrl: control; CBD: cannabidiol; ns: not significant; * *p* < 0.05, ** *p* < 0.01, **** *p* < 0.0001; *n* = 12 (three fields of view per sample).

Across all CBD concentrations and both timepoints (1 h and 24 h), the phenotype distribution of microglia in physiological M5 co-cultures remained unchanged, with no significant differences observed in either the homeostatic (RRT) or reactive (RPT) populations compared to controls. Thus, CBD did not significantly affect microglial phenotype or activation state in these cultures (Figure 2A–D).

In contrast, pathological M30 co-cultures showed significant, dose-dependent changes in microglial phenotypes in response to CBD (Figure 2E–H). After 1 h, ramified microglia increased from 65.2% in controls to 72.9% (500 ng/mL, *p* < 0.01) and 76.8% (1000 ng/mL, *p* < 0.0001), with reactive microglia accordingly decreasing. More pronounced effects were observed after 24 h: ramified microglia rose from 81.4% (control) to 92.5% (1000 ng/mL, *p* < 0.0001), with significant decreases in reactive microglia at all concentrations. These findings suggest that CBD promotes a homeostatic (“resting”) phenotype and suppresses activated microglia in M30 co-cultures in a concentration- and time-dependent manner.

#### 2.2.2. Glial Cell Numbers and Microglial Activation Were Not Affected by PGB Under Physiological and Pathological Conditions

**Figure 3:**
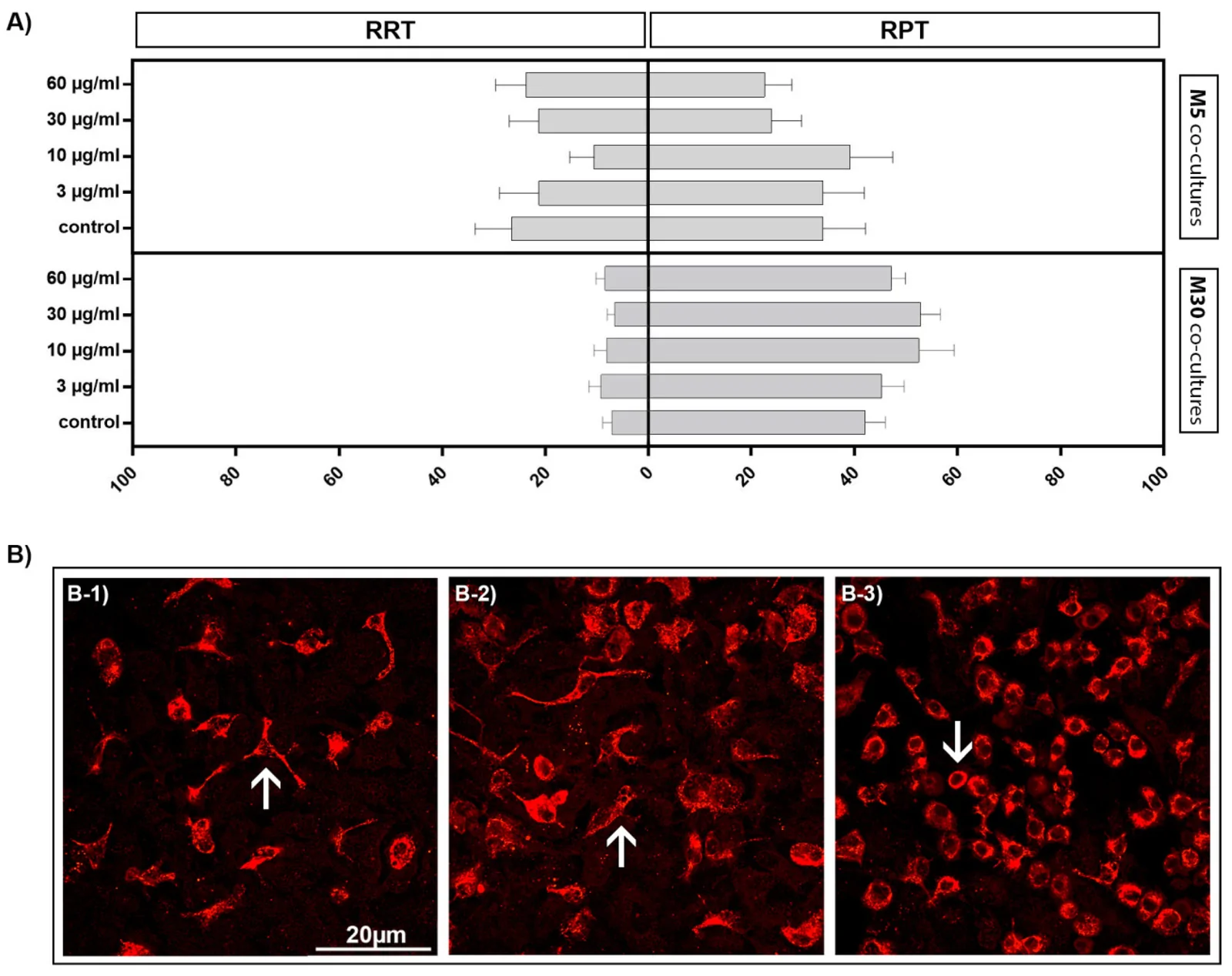
Immunocytochemical staining of microglial phenotypes allows classification as homeostatic ramified type (RRT) and rounded phagocytic type (RPT) in physiological M5 and pathological M30 co-cultures after concentration-dependent incubation with PGB (3, 10, 30 and 60 µg/mL) for 24 h. (**A**) Incubation of M5 (*n* = 24) and M30 (*n* = 16) co-cultures with different concentrations of PGB led to no significant changes in microglial phenotypes compared to controls. The D’Agostino–Pearson normality test and one-way analysis of variance (one-way ANOVA) followed by the Bonferroni post hoc comparison test were used for comparisons between the groups. Differences were considered significant at *p* < 0.05. Control: co-cultures treated with the vehicle PBS (24 µL per mL cell culture medium). (**B**) The microglial cells were labeled with the monoclonal antibody ED1 (red), allowing classification of all microglial phenotypes as homeostatic (“resting”) ramified (RRT) (**B-1**), intermediate (INT) (**B-2**) and activated rounded phagocytic (RPT) (**B-3**) phenotypes (white arrows) at a magnification of 600×. The RRT microglial phenotype is characterized by a reduction of the cell body and formation of long, thin processes longer than the diameter of the cell body (**B-1**); the INT microglia form only some thick cell processes whose size does not exceed that of the soma and possess a few vesicles and vacuoles in the cytoplasmic rim (**B-2**); the activated RPT microglial phenotype has a characteristically round soma with several cytoplasmic vacuoles and either rare short processes or no processes (**B-3**). A bar indicates 20 µm.

Incubation of the physiological M5 and pathological M30 co-cultures with PGB at different concentrations did not result in significant differences in the total number of cells or in the total number of microglia (Figure S5). The evaluation of the three microglial phenotypes was based on typical characteristics observed after immunocytochemical staining. Figure 3B illustrates individual ED1-stained microglial phenotypes: homeostatic (“resting”) ramified type (RRT) (Figure 3(B-1)), intermediate type (INT) (Figure 3(B-2)) and reactive round phagocytic type (RPT) (Figure 3(B-3)). Homeostatic ramified microglia are characterized by a reduction in the cell body and the formation of long, thin processes, whereas intermediate microglia form thick cell processes whose size does not exceed that of the soma. In the reactive form, microglia have a characteristically round soma without rami [34]. Evaluations based on these criteria showed that incubation with PGB in both physiological M5 and pathological M30 co-cultures did not lead to significant changes in microglial phenotypes (Figure 3A).

### 2.3. Effects of PGB and CBD on Cx43 Expression and Functional GJ Coupling

The Cx43 signal per cell detected by immunocytochemistry was not significantly affected by the incubation of physiological M5 and pathological M30 co-cultures with different concentrations of PGB for 24 h compared to untreated controls (Figure S6A–C). However, a tendency toward reduced Cx43 signal expression was observed in the M30 co-cultures (particularly at a concentration of 30 µg/mL PGB). In the M5 co-cultures, the average number of Cx43 signals per cell was 5.6 ± 2.5 (*n* = 18) (Figure S6A) in the control, while in the pathological M30 co-cultures, 4.6 ± 2.6 signals per cell (*n* = 17) (Figure S6B) were measured.

**Figure 4:**
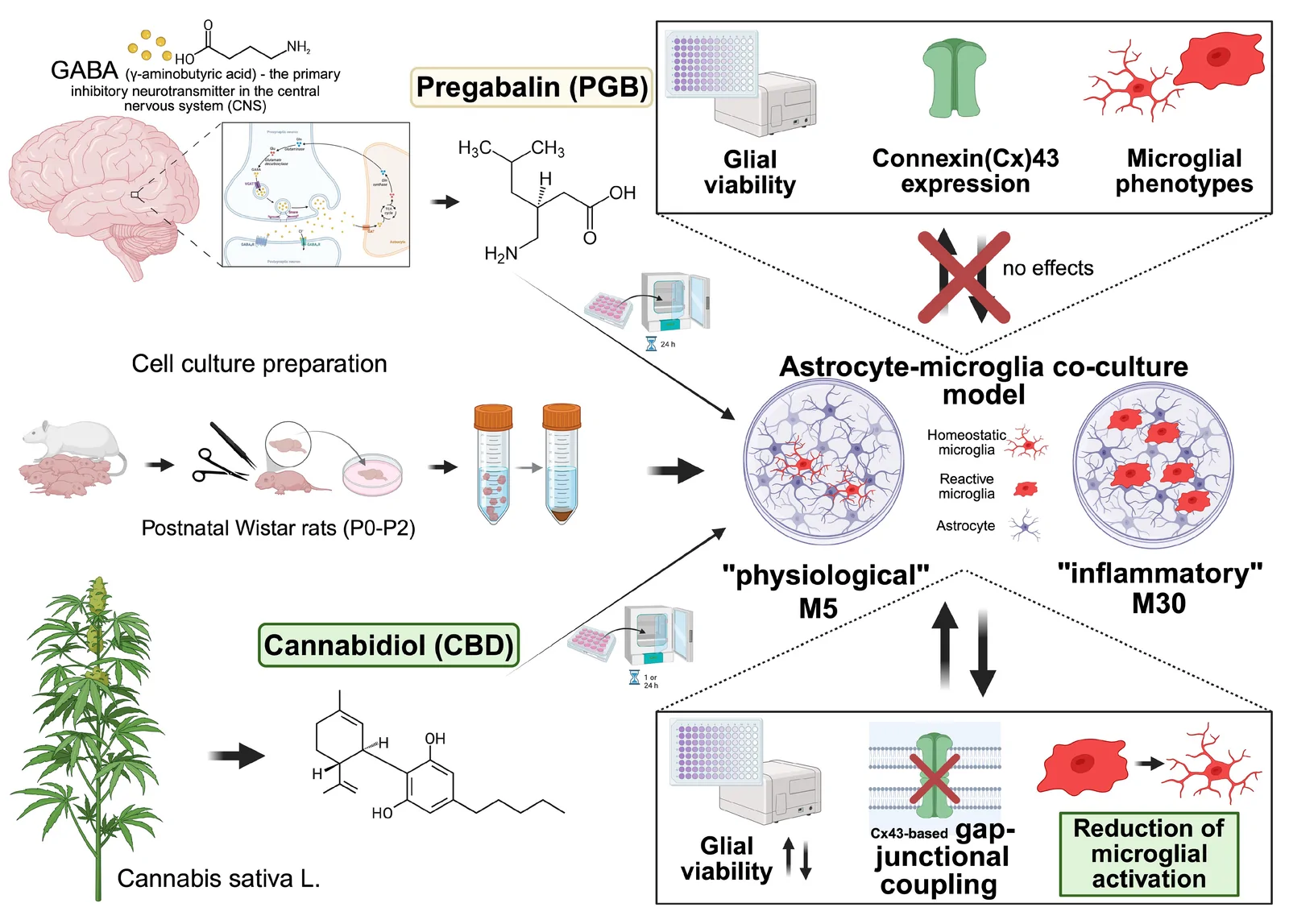
Overview of the key findings following the incubation of astrocyte–microglia co-cultures with PGB or CBD (graphical summary). Created in BioRender. Ismail, F. S. (2026) https://BioRender.com/sa1wtun (accessed on 23 July 2026).

In addition, the functional Cx43-mediated GJ coupling (measured using the scrape loading dye transfer method) in M5 and M30 co-cultures was not significantly affected by incubation with different concentrations of CBD for either one hour or 24 h (*n* = 12) (Figure S7). Figure 4 summarizes the key findings of the study.

## 3. Discussion

In summary, we can report effects of CBD on glial viability and microglial phenotypes in our astrocyte–microglia co-cultures, but no significant effects for PGB. For the physiological M5 cultures, which were incubated for one hour with CBD, negative effects on glial viability tended to be observed at the highest concentration. This is consistent with results from previous experiments with neural progenitor cells, which showed that dose-dependent neurotoxic effects can occur—a finding that is also partially reflected in the 24 h incubation with 1000 ng/mL CBD [63]. In contrast, with M5, an improvement in cell viability can even be observed at concentrations of 50 ng/mL and 500 ng/mL. We, therefore, suspect that CBD may have a stimulating effect at low doses, while higher doses have an inhibitory effect, as has also been observed with other active substances [64]. To verify the data, additional experiments using cell proliferation kits (e.g., with bromodeoxyuridine (BrdU)) or cell death markers associated with apoptosis or necrosis could be useful.

While the number of cells in M30 remained constant after incubation with CBD, a dose-dependent increase in cell numbers was observed in the M5 cultures. This is consistent with previous studies, in which repeated administration of CBD in in vitro cultures and in mice increased cell proliferation and cell cycle progression [65]. Another study also demonstrated that the administration of CBD increases the number of myeloid suppressor cells, which, like microglia, are of mesodermal origin [66]. On the other hand, the decrease in cell viability in the M5 cultures following treatment with 1000 ng/mL CBD could be attributed to possible dose-dependent toxicity [63]. This led to the question of why no changes were observed in the M30 cultures. One possible explanation is that the M30 cultures have already exhibited high baseline proliferation and microglial activity, meaning that any additional effects of CBD cannot be further amplified. Activated microglia tend to divide or initiate inflammatory processes. In such a state, further stimulus might not produce any visible differences. Furthermore, they might exhibit altered sensitivity to CBD. Activated microglia often undergo epigenetic changes or reprogramming, which could make them less responsive to proliferative stimuli [67].

The reduction of microglial reactivity by CBD, which may contribute to the anticonvulsant features, has been demonstrated in our astrocyte–microglia co-culture model. While the proportion of ramified microglia compared to activated microglia showed no changes in physiological M5 cultures following treatment with CBD, significant changes were observed in pathological M30 cultures. As the concentration increased, the proportion of activated microglia decreased, and the cells transitioned to a homeostatic state. In addition, the anti-inflammatory effects of CBD at the molecular level have been demonstrated in other studies. Possible molecular mechanisms may involve the modulation of cannabinoid receptors (CB1R, CB2R) or the inhibition of pro-inflammatory cytokines such as TNF-α or IL-6 by CBD. Previous studies suggested that the receptor plays a central role in anti-inflammatory effects [68,69]. We have demonstrated CB2 receptor expression in microglia in our co-cultures. However, further studies are needed to investigate the effects of CBD on receptor expression.

Overall, preclinical studies in chemically and electrically induced seizure and epilepsy models have consistently shown that CBD exerts anticonvulsant and neuroprotective effects, which are at least partially mediated by a reduction in neuroinflammation and glial cell activation. In models based on pentylenetetrazol and pilocarpine, as well as in temporal lobe epilepsy paradigms, CBD reduced seizure severity and epileptogenesis while limiting neuronal loss, accompanied by a shift in microglial polarization and reduced expression of pro-inflammatory cytokines such as IL-1β, IL-6, and tumor necrosis factor (TNF)-α in hippocampal networks. These findings, summarized in recent reviews and systematic analyses of CBD in experimental epilepsy, support a multimodal mechanism involving both endocannabinoid-related and additional signaling pathways that act on the balance between excitation and inhibition as well as neuroimmune regulation [14,16,19,20,21,70]. Our observation that, under pathological conditions in the microglia-astrocyte co-culture model, CBD promotes a transition from an activated to a more homeostatic microglial phenotype is therefore consistent with these in vivo data and extends them by providing a controlled glial cell system in which the anti-inflammatory and putative anticonvulsant effects of CBD can be investigated at the cellular level, thereby supporting and complementing the anticonvulsant mechanisms proposed in previous studies. Additional future studies are needed to systematically investigate inflammatory markers and key signaling pathways (e.g., NF-κB/STAT3 activation, cytokine and ROS profiles) in glial systems to more precisely define the glia-specific mechanisms of action of CBD in astrocytes and microglia and to clarify their contribution to the observed anti-inflammatory effects.

In this study, PGB did not affect the glial properties. PGB showed no effects on glial cell viability; even at high concentrations, PGB did not exert toxic actions. In addition, no changes in the total number of cells and microglia were determined. This is consistent with previous findings about the anti-apoptotic effects of PGB with regard to oligodendrocytes and neuroprotection in a rat model of spinal cord injury [71,72]. The mechanism of action of PGB is similar to the anticonvulsant drug GBT via binding to the alpha2-delta subunit of voltage-gated calcium channels [4]. In a previous study, GBT also did not affect glial viability in our physiological and pathological astrocyte–microglia co-cultures [54]. In contrast, a concentration-dependent incubation with other ASMs such as PHT, CBZ, LTG, and TPM led to possible cytotoxic effects on glial cells [54,58].

The involvement of glia-mediated inflammation in the pathogenesis of both epilepsy and neuropathic pain raises the question of how PGB may affect microglia in our astrocyte–microglia co-culture model of inflammation [45,46,48,49,50,51,52]. In contrast to previous studies showing anti-inflammatory features for PGB, in our in vitro model, no effects on microglia morphology were observed. Also, incubation with different concentrations of GBT for 24 h caused no significant alteration of microglial activation in our astrocyte–microglia co-culture model [54]. In animal models of MS, referred to as experimental autoimmune encephalomyelitis, PGB led to attenuation of astrogliosis and microglial reaction by reducing the expression of activated glial markers GFAP and Iba1 [73,74]. In a rat model of spinal cord injury, PGB reduced microglial activation, suggesting anti-inflammatory effects [71,72]. In lipopolysaccharide/concanavalin A-induced murine models of inflammation, PGB inhibited the secretion of the pro-inflammatory cytokines IL-6, TNF-α and IL-2 in splenocytes in vitro [75]. In vivo, PGB also inhibited the pro-inflammatory IL-6, TNF-α, IL-1β and IL-2 secretion. However, PGB showed no effects on cytokine secretion in peritoneal macrophages in vitro [75], which is consistent with our results about the lack of effects on microglia phenotypes in our in vitro co-culture model. These points are interesting for pain treatment during the course of inflammatory CNS diseases such as MS with spinal cord involvement because PGB could provide benefits due to missing inflammation-enhancing effects. In neuropathic rats, treatment with PGB led to up-regulation of anti-inflammatory IL-10 and analgesic β-endorphin mRNA and protein expression in primary spinal microglia, but not astrocytic or neuronal cells. These effects were related to the attenuation of neuropathic pain [76]. Thus, the results of in vitro and in vivo studies regarding PGB effects on glia-mediated inflammation are divergent. A possible explanation could be the different study designs. In conclusion, additional studies regarding further microglial activation markers and cytokines in the astrocyte–microglia co-culture model could be useful.

Increased Cx43 expression and GJC between astrocytes were related to increased seizure activity [39,40,41,77,78]. Moreover, astrocytic Cx43 was linked to the development of central neuropathic pain following spinal cord injury, supporting a crucial role of astrocytes in the development of chronic pain [42,43,44]. There are no previous data about PGB effects on Cx43 expression available. In our co-culture model, incubation with different concentrations of PGB did not alter the Cx43 expression under physiological and pathological conditions. Similar to these findings, GBT also did not alter Cx43 protein levels in our co-culture model [54]. Faustmann et al. (2003) showed that microglial activation may influence the Cx43 expression and functional coupling in the astrocytic network [34]. In line with these findings, incubation with PGB led to no alterations of microglial activation states or Cx43 expression. Further studies investigating Cx43 expression by additional methods such as Western blot and GJ coupling may contribute to a better understanding of PGB effects on the astrocytic network. Another limitation of our study is that, although we examined different concentrations of PGB, we used only one incubation time (24 h). Therefore, it remains unclear whether shorter or longer incubation with PGB could have effects on glial cells. But based on previous experimental studies, our incubation time (24 h) is assumed to be sufficient to study the drug effects.

CBD did not significantly affect GJ coupling in either M5 or M30 cultures. Taken together, these findings indicate that CBD did not significantly alter GJ coupling under the conditions tested, but they do not exclude a subtle, dose-dependent modulation in pathological M30 cultures. However, in the M30 cultures that were incubated with CBD for 24 h, a dose-dependent but non-significant reduction in Lucifer yellow dye transfer was observed. Similar results were observed in another study with endothelial cells, where cannabinoids inhibited communication via GJs [79]. Similar mechanisms may be at work in astrocytes, albeit more likely at high doses. Although the current results do not show significant effects, the observation in the M30 cultures underscores the need for further investigation. The dose-dependent decrease in communication could indicate subtle changes in Cx43 expression or function. Cx43 is known to be dynamically regulated, particularly under pathological conditions such as those present in the M30 cultures. Future investigations using Western blot and qPCR to quantify possible dose-dependent effects of CBD are needed.

Although our findings support a CBD-induced shift toward a more homeostatic microglial phenotype, the present study is limited by the lack of additional inflammatory biomarkers and signaling analyses that would further strengthen the mechanistic interpretation. Future studies should include analyses of intracellular signaling pathways, such as NF-κB- or STAT3-related cascades, to better define the molecular basis of CBD-induced changes in microglial activation. Complementary immunophenotyping and cytokine profiling may further help distinguish direct effects on microglial reactivity from broader glial responses. Likewise, additional astrocyte-specific functional assays, such as glutamate uptake or calcium signaling, would be valuable to complement the present Cx43-related analysis.

Moreover, only a 24 h incubation period for PGB was examined in the present study, which may not capture slower or delayed drug effects. In addition, the MTT assay reflects metabolic activity rather than proliferation alone; thus, the observed MTT changes cannot be unequivocally attributed to increased proliferation, increased metabolic activity per cell, or both. Complementary assays such as BrdU incorporation and apoptosis markers will be required in future studies.

In addition, no separate LPS-induced inflammatory condition was tested, as the M30 co-culture was used as an established inflammatory baseline model. Future studies could combine this system with exogenous inflammatory triggers to further dissect CBD-related mechanisms. Taken together, these limitations also underscore that the present findings represent preclinical evidence from a rat in vitro model. Further studies in human-derived systems and in vivo settings will therefore be necessary to determine the extent to which these observations translate to human biology.

## 4. Materials and Methods

### 4.1. Cell Culture

The cerebral hemispheres of Wistar rats were used to produce astrocyte–microglia co-cultures, which were established as a glial inflammation model by Faustmann et al. (2003) and have since been widely used in other pharmacological studies [34,54,55,56,57]. Wistar rats (wild type) used for astrocyte co-cultures were bred and housed at the Central Animal Facility of the Medical Faculty at Ruhr University Bochum, Germany. In Germany, the use of vertebrate animals for scientific purposes is governed by the Animal Welfare Act. The institutional Animal Welfare Officer ensures compliance with national and EU animal welfare regulations.

According to German legislation (Animal Welfare Act) and European regulations, the collection of organs or cells from vertebrate animals is not classified as an animal experiment, provided that no surgical or invasive procedures are carried out before euthanasia. The postnatal rats in our study were euthanized by decapitation before brain tissue was removed, and no surgical or invasive procedures were performed prior to euthanasia. Based on these legal definitions, ethical approval is not necessary for this procedure. The Animal Welfare Officer of Ruhr University Bochum confirmed that the experimental protocols used in the research project for generating primary cell cultures have been approved as being in accordance with animal protection regulations. Furthermore, he confirmed that the housing and husbandry of the experimental animals used in the research project were in accordance with all applicable legal regulations and that personnel and housing facilities had the necessary official permissions. In summary, all protocols used to generate primary cell cultures were conducted in accordance with animal welfare guidelines.

At the beginning of the experiment, postnatal day 0–2 (P0–P2) Wistar rats (*n* = 20) were euthanized by decapitation. The cranium was then opened to allow the removal of the two hemispheres. The isolated brain hemispheres were immediately transferred to ice-cold phosphate-buffered saline (PBS) (containing 1.38 M NaCl, 27 mM KCl, 81 mM Na_2_HPO_4_, 14.7 mM KH_2_PO_4_) (Roth, Karlsruhe, Germany; J.T. Baker, Deventer, The Netherlands; Merck, Darmstadt, Germany; VWR, Radnor, PA, USA) to ensure optimal preservation of tissue integrity during removal of choroid plexus and meninges. The hemispheres were then transferred into warm PBS.

For approximately 10–14 hemispheres, 450 μL of 2.5% trypsin solution (Thermo Fisher Scientific, Dreiech, Germany) were pipetted into a 50 mL Falcon tube containing 10 mL of warm PBS. The cerebri were then transferred, mechanically dissociated with a pipette, and incubated in a water bath at 37 °C for 30 min. The homogenate was then centrifuged for 7 min at 500 g and room temperature (RT), and the trypsin-PBS supernatant was discarded. Next, 4 mL of sterile astrocyte medium and 1 mL of DNAse (Serva Electrophoresis, Heidelberg, Germany) was added to the pellet and mixed. After incubating for 5 min at RT, the mixture was filled to 20 mL with sterile washing medium and centrifuged for another 5 min at 200 g and RT. This process was repeated once more.

The pellet was resuspended in 4 mL of sterile astrocyte medium. The suspension was then filtered through a sterile nylon filter with a pore size of 60 μm to remove the larger cells, such as erythrocytes. Next, 5 mL of medium was added to each of two to four culture bottles, and the filtered suspension was distributed evenly. The cells were then incubated at 37 °C and 5% CO_2_ for 24 h. The volume of 200 mL of astrocyte medium consisted of 174 mL of Dulbecco’s minimal essential medium, DMEM (Thermo Fisher Scientific, Dreiech, Germany), 20 mL of fetal calf serum, FCS (PAN Biotech, Aidenbach, Germany), and 2 mL each of non-essential amino acids (HyClone, Cytiva, Marlborough, MA, USA), glutamine (GE-Healthcare, Munich, Germany) and penicillin/streptomycin (Thermo Fisher Scientific, Dreiech, Germany/PAA Laboratories, Linz, Austria). The washing medium did not contain glutamine or amino acids.

The culture was washed again the next day, and after regular visual inspection of the cells, the medium was changed every 3 d. Passaging was performed when confluent growth was achieved. Depending on shaking following culture preparation, the proportion of microglial cells in the co-cultures varied and was determined by immunocytochemical staining. Shaking (1 h at 300 rpm at RT) led to a reduction in the number of microglia, since microglia are predominantly located above layers of astrocytes and, as a result of shaking, can be detached and removed with the cell culture medium, resulting in the establishment of M5 conditions [34,54,80]. In the established inflammatory co-culture model described by Hinkerohe et al., cultures containing approximately 5% microglia (M5) represent the physiological condition, whereas cultures containing approximately 30% microglia (M30) represent the inflammatory condition. This model was originally validated by Hinkerohe et al. through cytokine-induced changes in microglial phenotype, including TNF-α, IL-1β, IL-6, IFN-γ, and TGF-β1, astroglial connexin 43 expression, functional coupling, and membrane resting potential. These published criteria based on inflammatory markers were used here to define the M5 and M30 conditions [80]. For example, incubation with the pro-inflammatory cytokines TNF-α, IL-1β, IL-6, and IFN-γ induced microglial activation in M5 co-cultures that is comparable to the conditions in M30 co-cultures. Furthermore, incubation with the anti-inflammatory TGF-β1 was able to reduce microglial activation in M30, while IFN-β was able to prevent the microglial activation induced by pro-inflammatory cytokines in M5 [80]. In another study by Hinkerohe et al., in control co-culture supernatants corresponding to M5 conditions (median microglia content of 5%), low concentrations of the cytokines TNF-α, IL-1β, IL-10 and IFN-γ were detected (median: 9.31 pg/mL TNF-α, 6.08 pg/mL IL-10, < 0 pg/mL IL-1β and 3.90 pg/mL IFN-γ). After incubation of the control M5 co-cultures with LPS, the concentrations of the cytokines, particularly TNF-α and IL-1β, as well as the percentage of activated microglial phenotype, significantly increased [81].

During the course of the experiments, four independent culture preparations were carried out, and the cultures were passaged up to three times when they reached approximately 90–100% confluency.

### 4.2. Treatment of Cells

The concentration ranges of PGB were chosen according to previous experimental reports and therapeutic concentrations in humans [75,82,83,84]. The astrocyte–microglia co-cultures M5 (representing the physiological state) and M30 (representing the pathological state) were incubated with different concentrations (3, 10, 30 and 60 µg/mL) of PGB (Cayman Chemical, Ann Arbor, MI, USA) in 5% CO_2_ at 37 °C for 24 h. The concentrations of 3–10 µg/mL correspond to the therapeutic range [82]. The high concentrations (30 and 60 μg/mL) were selected to study the cytotoxic effects of PGB overdose. The drug was dissolved in PBS (2.5 mg/mL) and diluted in the culture medium. The control co-cultures were treated with the vehicle PBS (24 µL per mL of cell culture medium).

Treatment with different concentrations of CBD (50 ng/mL, 500 ng/mL, or 1000 ng/mL) (THC Pharma, Frankfurt, Germany) was also performed in the astrocyte–microglia co-cultures in either a pathological state (M30) or a physiological state (M5) at 37 °C and 5% CO_2_ for one or 24 h. Previous studies showed that seizure control is proportional to the CBD plasma level (ranging from 7.1–1200 ng/mL). Correspondingly, CBD concentrations of 50, 500 and 1000 ng/mL were chosen in our study to evaluate therapeutic and possible overdose effects [85,86]. Due to the hydrophobic properties of CBD, the corresponding amounts were first dissolved in DMSO (Thermo Fisher Scientific, Dreieich, Germany). The control co-cultures were incubated with 0,1% DMSO, which corresponded to the highest concentration used to dissolve CBD for the treatment of the cells. The DMSO concentration needs to be less than 1% of the medium so as not to damage the astrocytes.

### 4.3. MTT Assay

To evaluate proliferation, vitality, and cytotoxicity in astrocyte–microglia cultures after incubation with PGB or CBD, an MTT assay (3-(4,5-dimethylthiazol-2-yl)-2,5-diphenyltetrazolium bromide, Roche Applied Sciences, Prenzberg, Germany) was performed. Cell viability was evaluated based on mitochondrial activity. Living cells convert the MTT reagent, a tetrazolium salt, into an insoluble formazan dye, which enables the quantification of living cells.

In preparation for the MTT assay, astrocyte–microglia co-cultures were seeded at a density of 20,000 cells per well. Four separate 96 well plates were prepared in total. On each plate, wells were allocated to the vehicle control and the respective treatment concentrations under standardized seeding conditions. Each condition was measured across 24 wells on the respective plate. Cultivation of the microtiter plates was performed until subconfluence of the cultures at 37 °C and 5% CO_2_ in the incubator. Following this, the co-cultures were incubated with PGB or CBD as previously described. After the incubation period, 10 μL of MTT reagent was added to each well to start the MTT assay. This was followed by a four-hour incubation at 37 °C and 5% CO_2_. Subsequently, 100 μL of solubilization solution was added to each well, and the samples were incubated overnight. After 24 h, the optical density was measured at 550 nm using a microplate reader (Infinite M Nano+, Tecan Trading AG, Männedorf, Switzerland). The results were statistically evaluated using GraphPad Prism 10.4.0.

### 4.4. Immunocytochemistry

Immunocytochemistry was used to visualize microglial phenotypes and cell numbers. In a healthy brain, i.e., under physiological conditions, the primary phenotype is the homeostatic (previously known as “resting”) ramified type (RRT). This type is characterized by a small round cell body with long, thin branches. The second type is the reactive phagocytic type (RPT), which has few short or no processes. In contrast to homeostatic microglia, the reactive type is able to move in order to respond to signals such as foreign bodies [34,67].

For immunocytochemistry, poly-L-lysine (PLL)-coated coverslips (12 mm) were placed in the wells of a 24 well plate. Then, 70,000 cells per well were added. This was followed by a 96 h incubation at 37 °C and 5% CO_2_ to achieve at least 70% subconfluence. After that, the cultures were incubated with PGB, CBD or the control medium for one or 24 h. Following incubation, coverslips were washed three times each with 1 mL PBS for 5 min and fixed in 1 mL of ice-cold ethanol (100%) for 10 min. Afterwards, they were washed again three times with PBS.

Next, a blocking solution consisting of PBS with 1% bovine serum albumin (BSA) (AppliChem, Darmstadt, Germany) and 10% horse serum (Thermo Fisher Scientific, Dreiech, Germany/PAA Laboratories, Linz, Austria) was added to the cells for one hour to block non-specific binding sites for the antibodies. The primary monoclonal antibody (mouse) anti-ED1 (anti-CD68 microglia/macrophage marker) (1:250) (Bio-Rad Laboratories, Feldkirchen, Germany/Serotec, Düsseldorf, Germany), the primary polyclonal antibody (rabbit) anti-CNR/CB2 (cannabinoid receptor 2) (1:250) (Bioss Antibodies, Woburn, MA, USA), and the primary monoclonal antibody (rabbit) anti-Cx43 (1:1000) (Invitrogen, Karlsruhe, Germany) were prepared in PBS-blocking solution, and 25 μL was added to each coverslip. The incubation took place overnight at 4 °C in a humid chamber.

The following day, the cultures were washed three times for 15 min with a 1% BSA/PBS solution. Next, the secondary antibodies Alexa Fluor^®^ 568 red (mouse) (1:500) and Alexa Fluor^®^ 488 green (rabbit) (1:500) (Invitrogen, Karlsruhe, Germany) were diluted in PBS-blocking solution, and 25 μL was added to each culture. After incubating in the dark for one hour, the cultures were washed three times for 10 min with PBS. Next, 3–4 μL of mounting medium ProLong Gold DAPI (Thermo Fisher Scientific, Dreieich, Germany/Invitrogen, Karlsruhe, Germany), which contains the nuclear stain DAPI (4′,6-diamidino-2-phenylindole), was added to each coverslip. The coverslips were stored at 4 °C in the dark until further use.

The evaluation was performed using the Leica DM IL LED inverted light microscope (Leica Microsystems GmbH, Wetzlar, Germany) at 400× magnification or the Zeiss Axiovert 100M laser scanning confocal microscope (Carl Zeiss, Jena, Germany) at 600× magnification. For all immunocytochemistry analyses, at least three different visual fields were randomly selected by coverslip for quantification. The images were then manually counted to determine the total number of cells and the number of microglia. In addition, the microglial cells were classified according to optical criteria by immunocytochemistry. Reactive microglia (RPT type) are characterized by round cell bodies without cell processes, while homeostatic (“resting”) microglia (RRT type) have long thin processes (examples of microglial phenotypes are shown in the Section 2, Figure 3). The ImageJ software program (version 1.53v) was used to determine the immunocytochemical Cx43 signal per cell (Rasband, W.S. ImageJ. National Institutes of Health, Bethesda, MD, USA).

### 4.5. Scrape Loading and Dye Transfer

The scrape loading dye transfer method enabled the investigation of functional GJ coupling based on the properties of the fluorescent dye Lucifer yellow, which cannot pass through intact cell membranes but can be transported through GJ (mainly Cx43-based in astrocytes) when the cells are coupled by previously damaged membranes. For the experiment, the cells were seeded on 24 well plates (100,000 cells per well). After reaching 100% confluence, the M5 and M30 co-cultures were incubated with CBD for one or 24 h as described above. Following washing with PBS, 400 µL of 0.03% (*w*/*v*) Lucifer yellow CH solution (in PBS) (Invitrogen, Karlsruhe, Germany) was added to each well, and a linear incision was made on the confluent cell surface with an injection needle (0.45 × 12 mm) (Braun, Melsungen, Germany). The co-cultures were incubated for 2 min at 37 °C and 5% CO_2_ in the dark and were then washed with PBS again. The evaluation of the GJ coupling was performed immediately using the Leica DM IL LED inverted light microscope (Leica Microsystems GmbH, Wetzlar, Germany) at an excitation wavelength of 488 nm and 100× magnification. The percentage value of Lucifer yellow fluorescent intensity was evaluated using the ImageJ software program (version 1.53v) (Rasband, W.S. ImageJ. National Institutes of Health, Bethesda, MD, USA) in three randomized visual fields per coverslip related to the incision site.

### 4.6. Data Analysis and Statistics

Data analysis and statistics were carried out using GraphPad Prism 7.0/10.4.0 for Windows (GraphPad Software, San Diego, CA, USA). D’Agostino-Pearson omnibus tests were performed to analyze the normality of data distribution. In cases where normality was given, parametric tests were used. One-way analysis of variance (one-way ANOVA) followed by the Kruskal–Wallis test or Bonferroni post hoc comparison test was performed to analyze comparisons between more than two groups with normal distribution, or followed by Dunn’s test for non-parametric data. The significance was set at *p* < 0.05. The results were presented as mean ± standard error of the mean, reflecting the precision of the estimated group mean across independent experiments.

## 5. Conclusions

The inhibition of microglial reactivity by CBD indicates potential effects on the neuroinflammatory component involved in the pathogenesis of CNS disorders such as epilepsy and MS. By comparison, the present study indicates that the mechanisms of action of PGB probably do not include modulation of glial cells in vitro. Specifically, the microglial phenotypes were not affected by PGB in our co-culture model.

## Data Availability

The raw data supporting the conclusions of this article are available upon request to the corresponding author.

## Supplementary Materials

The following supporting information can be downloaded at: https://www.mdpi.com/article/10.3390/ph19091342/s1. Figure S1. Glial cell viability after concentration-dependent incubation with pregabalin (PGB); Figure S2. Total cell numbers in M5 and M30 co-cultures after incubation with various concentrations of cannabidiol (CBD); Figure S3. Total microglial cell numbers in M5 and M30 co-cultures after cannabidiol (CBD) incubation; Figure S4. Immunocytochemistry of cannabinoid receptor 2 (CNR/CB2) expression in microglia; Figure S5. The total number of glial cells and microglia after concentration-dependent incubation with pregabalin (PGB); Figure S6. Connexin 43 (Cx43) signal per cell after concentration-dependent incubation with pregabalin (PGB); Figure S7. Functional gap-junctional coupling in M5 and M30 astrocyte-microglia co-cultures after incubation with different concentrations of cannabidiol (CBD).
