# Supplementary material for: Reduction of Microglial Reactivity by Cannabidiol: Preliminary Data Obtained in an Astrocyte–Microglia Co-Culture Model of Inflammation
Source: Pharmaceuticals (Basel). 2026 Aug 24;19(9):1342. doi: 10.3390/ph19091342 (PMC13610370; doi:10.3390/ph19091342)
Supplement: Supplementary file 1 [file pharmaceuticals-19-01342-s001.zip › pharmaceuticals-4456754-supplementary.pdf]

Supplementary Materials (Figures)

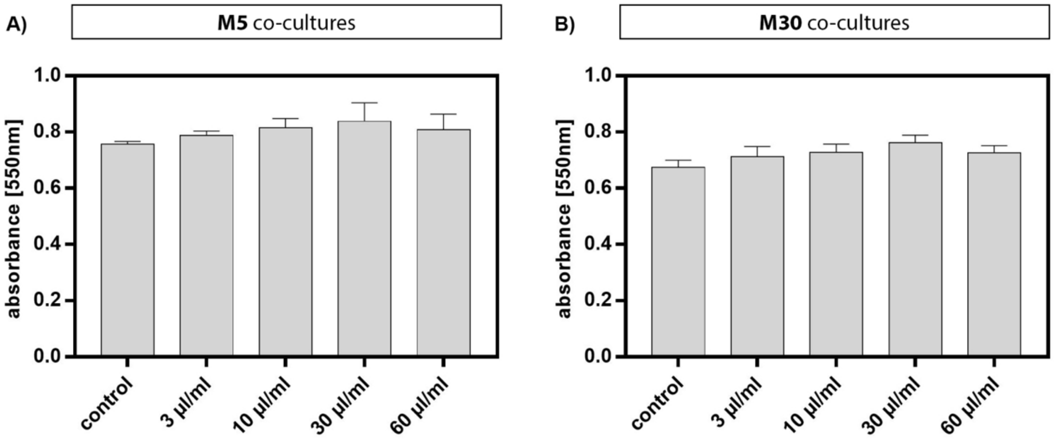

Figure S1. Glial cell viability was measured by MTT assay in physiological M5 **(A)** and pathological M30 **(B)** astrocyte–microglia co-cultures after concentration-dependent incubation with pregabalin (PGB) (3, 10, 30 and 60 µg/mL) for 24 h. The glial cell viability was not affected by PGB under both conditions compared to controls (M5 $n = 8$; M30 $n = 16$) **(A, B)**. The D'Agostino–Pearson normality test and one-way analysis of variance (one-way ANOVA), followed by Bonferroni post hoc comparison test, were used for comparisons between the groups. Differences were considered significant at $p < 0.05$. Control: co-cultures treated with the vehicle PBS (24 µL per ml of cell culture medium).

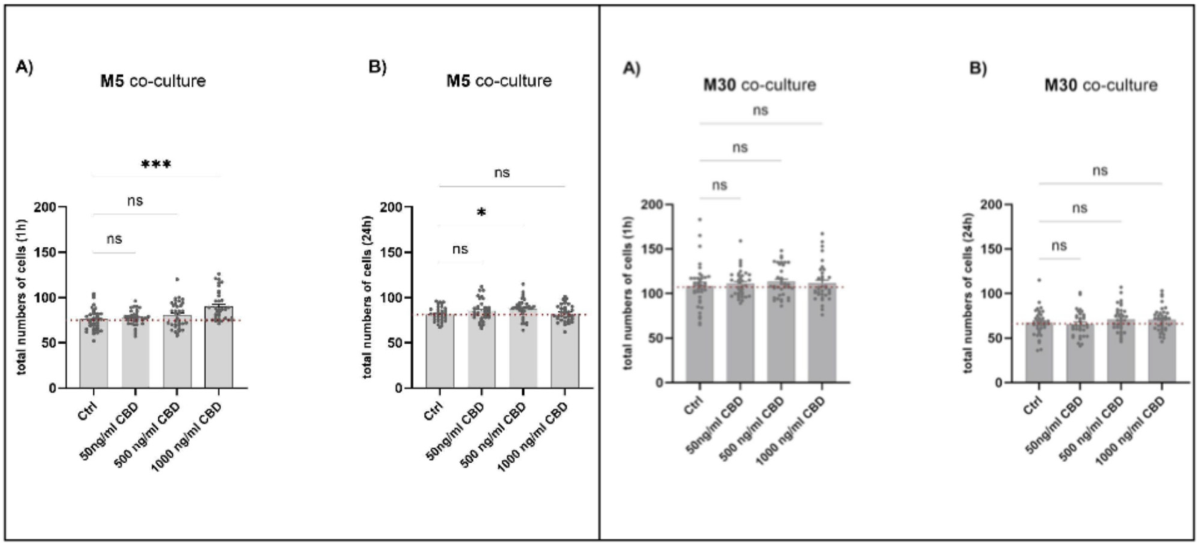

Figure S2. Total cell numbers in M5 and M30 co-cultures after incubation with various concentrations of cannabidiol (CBD) for 1 or 24 h, quantified using immunocytochemistry (DAPI). (**A**) 1 h and (**B**) 24 h results for both models are shown. In M5 co-cultures, the cell number increased significantly for 1000 ng/mL CBD at 1 h ($p < 0.001$) and for 500 ng/mL CBD at 24 h ($p < 0.05$), while all other conditions were unchanged. No significant differences were observed in M30 co-cultures at any time or concentration. Ctrl: control; CBD: cannabidiol. Statistics: one-way ANOVA, Kruskal–Wallis test; ns: not significant; *$p < 0.05$, **$p < 0.01$, ***$p < 0.001$, ****$p < 0.0001$; $n = 12$, three fields of view per sample.

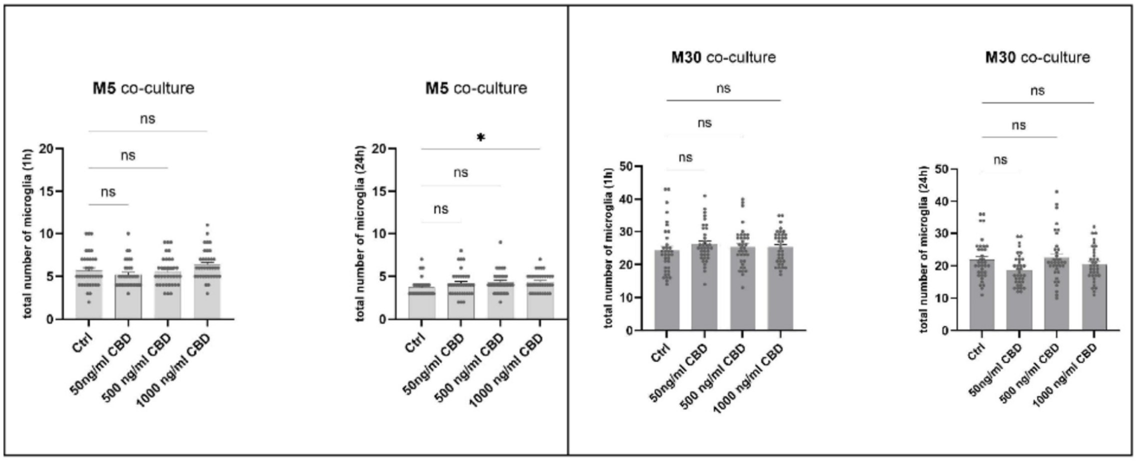

Figure S3. Total microglial cell numbers in M5 and M30 co-cultures after cannabidiol (CBD) incubation for 1 h and 24 h, quantified by ED-1 immunostaining. In M5 co-cultures, microglial numbers remained stable at 1 h, but increased significantly after 24 h with 1000 ng/mL CBD ($p < 0.05$). In M30 co-cultures, microglial counts were unaffected by CBD at either time point or concentration. Ctrl: control; CBD: cannabidiol. Statistics: one-way ANOVA, Kruskal–Wallis test; ns: not significant; *$p < 0.05$, **$p < 0.01$, ***$p < 0.001$, ****$p < 0.0001$; $n = 12$, three fields of view per sample.

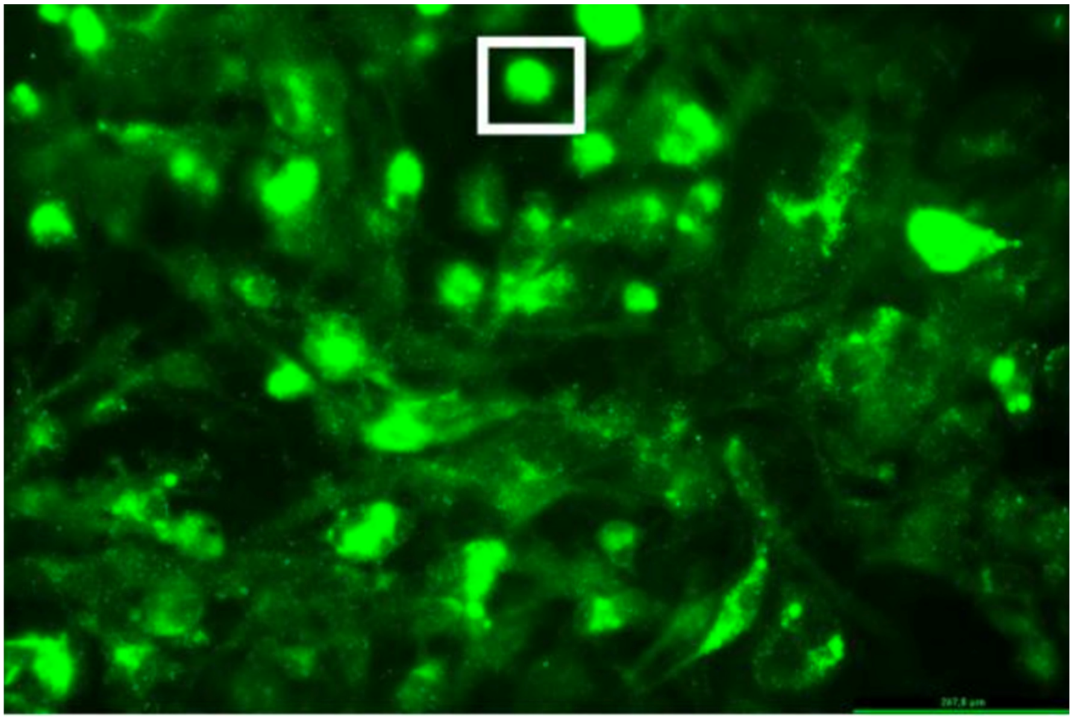

Figure S4. Immunocytochemistry of cannabinoid receptor 2 (CNR/CB2) expression (green) in microglia (example in M30 co-cultures with a reactive rounded phagocytic phenotype (RPT) of microglia, indicated by the white frame). The evaluation was performed using the Leica DM IL LED inverted light microscope (Leica Microsystems GmbH, Wetzlar, Germany) at 400× magnification.

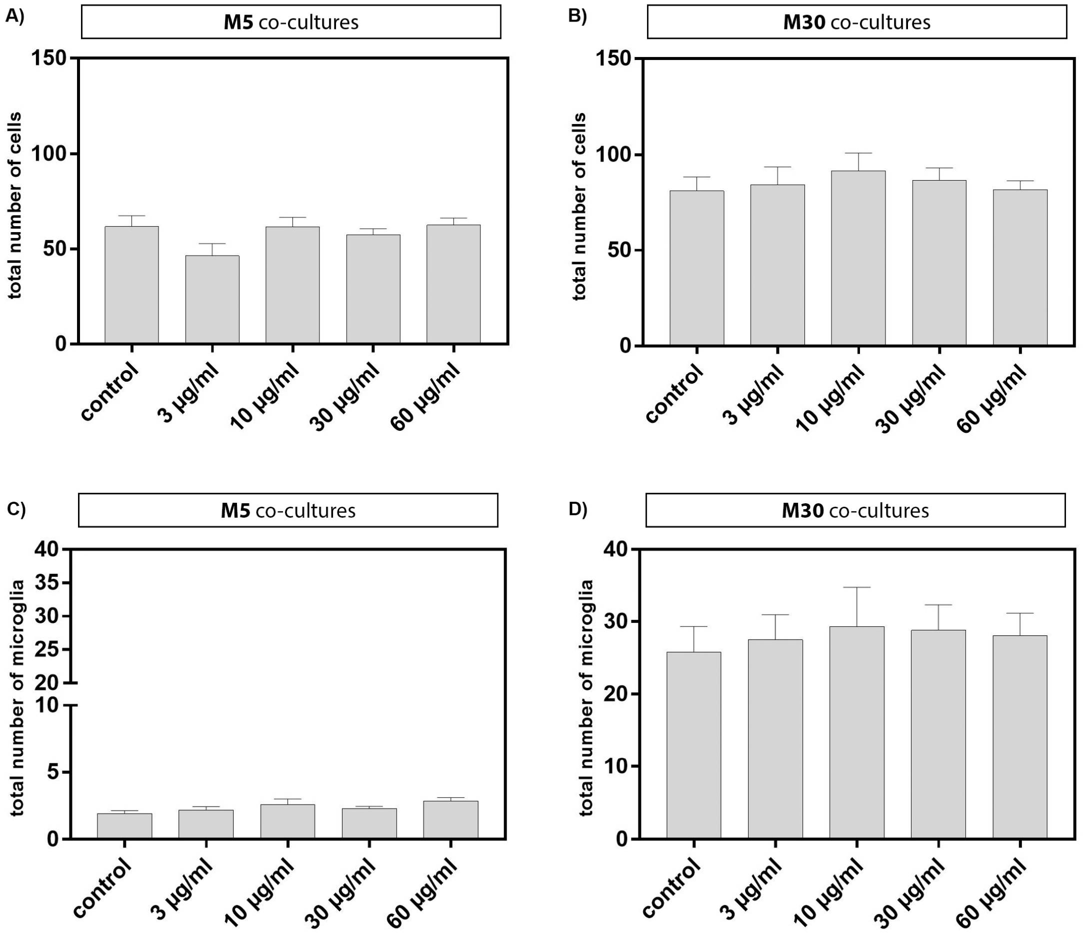

Figure S5. The total number of glial cells and microglia was detected by immunocytochemistry in physiological (M5; **A, C**) and pathological (M30; **B, D**) astrocyte–microglia co-cultures after concentration-dependent incubation with pregabalin (PGB) (3, 10, 30 and 60 µg/mL) for 24 h. No significant changes were detected after incubation with PGB for both the total number and amount of microglia (n = 16, n = 26). The D'Agostino–Pearson normality test and one-way analysis of variance (one-way ANOVA), followed by the Bonferroni post hoc comparison test, were used for comparisons between the groups. Differences were considered significant at $p < 0.05$. Control: co-cultures treated with the vehicle PBS (24 µL per ml of cell culture medium).

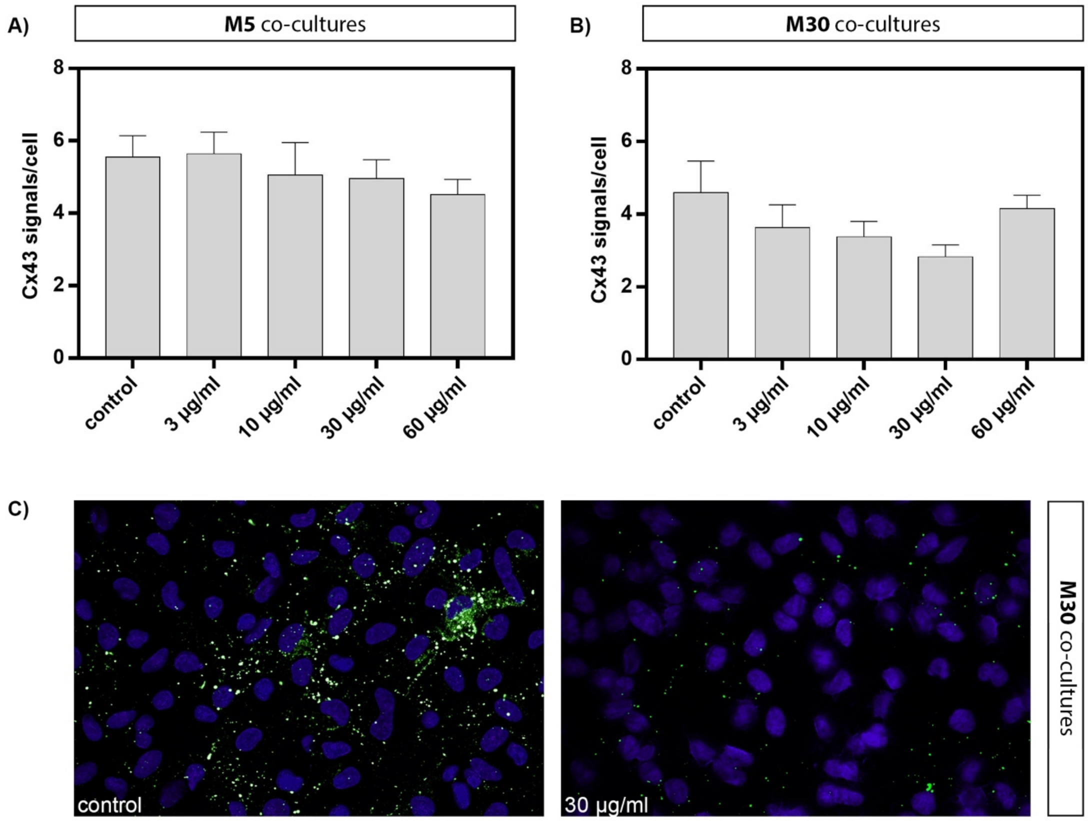

Figure S6. Connexin 43 (Cx43) signal per cell was identified by immunocytochemistry after concentration-dependent incubation with pregabalin (PGB) (3, 10, 30 and 60 μg/mL) for 24 h in physiological M5 ($n = 18$) **(A)** and pathological M30 ($n = 17$) **(B)** astrocyte–microglia co-cultures, resulting in no significant changes. However, there was a tendency toward a reduction in Cx43 signal expression in M30 co-cultures. The panels in **(C)** show exemplary anti-Cx43 (green) and DAPI (blue, cell nuclei) stained cells of M30 co-cultures incubated with 30 μg/mL PGB and the corresponding control. The evaluation was performed using the Zeiss Axiovert 100M laser scanning confocal microscope (Carl Zeiss, Jena, Germany) at 600× magnification. The D'Agostino–Pearson normality test and one-way analysis of variance (one-way ANOVA), followed by the Bonferroni post hoc comparison test, were used to compare the groups. Differences were considered significant at $p < 0.05$. Control: co-cultures treated with the vehicle PBS (24 μL per ml of cell culture medium).

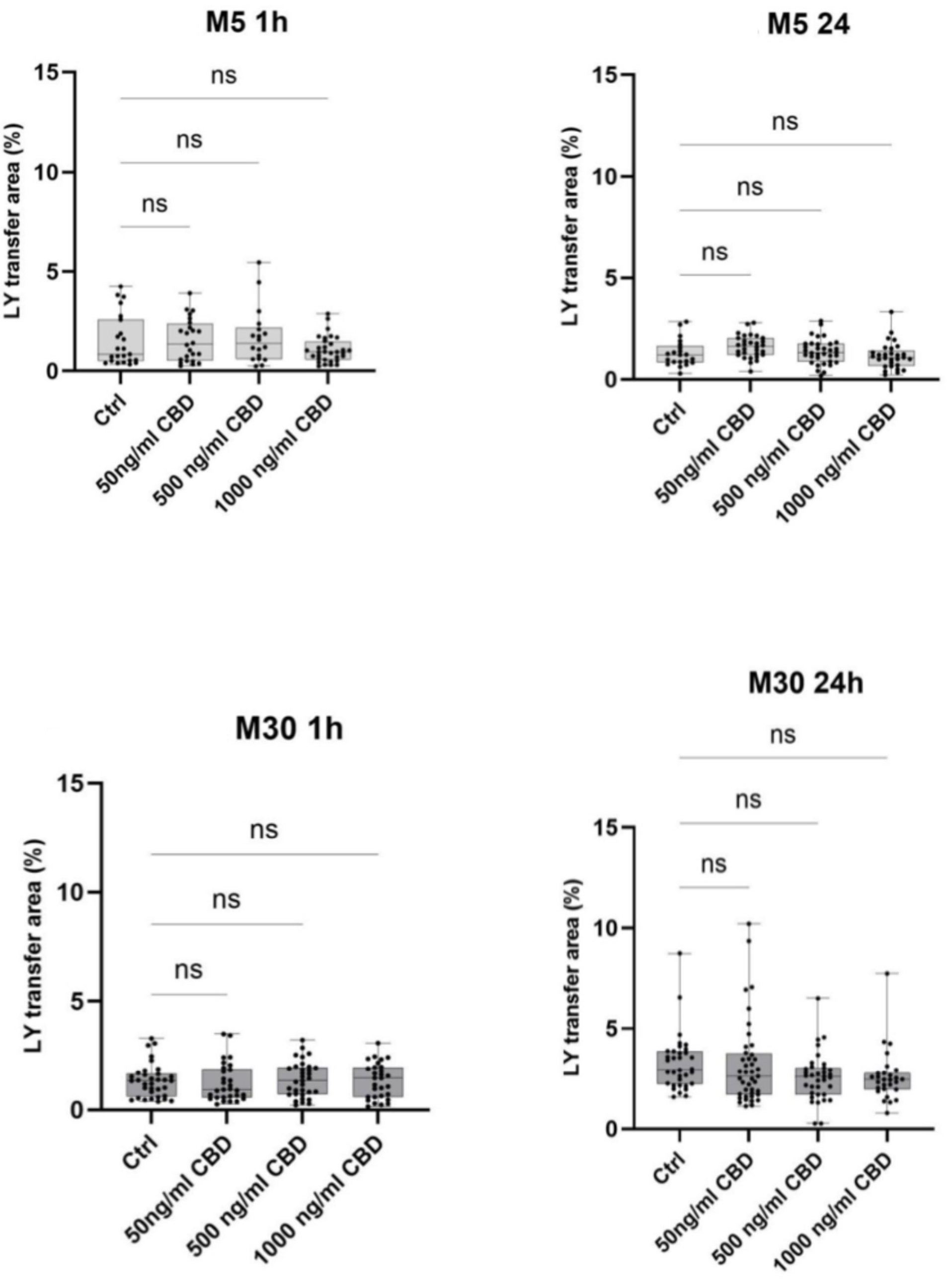

Figure S7. Functional gap-junctional coupling in M5 and M30 astrocyte–microglia co-cultures after incubation with different concentrations of cannabidiol (CBD) for 1 or 24 h. The percentage value of Lucifer yellow (LY) fluorescent intensity was evaluated using the ImageJ software program (version 1.53v) (Rasband, W.S. ImageJ. National Institutes of Health, Bethesda, Maryland, USA). No significant differences were observed in M5 and M30 co-cultures at any time

or concentration. Ctrl: control; CBD: cannabidiol. Statistics: one-way ANOVA, Kruskal–Wallis test; ns: not significant; *$p < 0.05$, **$p < 0.01$, ***$p < 0.001$, ****$p < 0.0001$; $n = 12$, three fields of view per sample.
